# Hospital and Institutional News

**Published:** 1920-10-30

**Authors:** 


### HOSPITAL AND INSTITUTIONAL NEWS.

### THE MINISTRY OF HEALTH.

According to *The Times* this week a very angry storm is brewing against the Ministry of Health, and to whatever extent this may be true, the medical correspondent to our contemporary does not proceed to pour oil on the troubled waters. Instead he hits vigorously right and left, and is apparently more than exasperated with committees, their talks, and reports generally. We heartily agree that health cannot come by statute, but, nevertheless, public-health work, in its new sense, requires no little consideration, be we dealing with hospital systems on the large scale or the size of local panel practices. Also we must admit that the findings of certain committees are, so far as they have yet gone, somewhat inconclusive to say the least of it. There is ample room for destructive criticisms, but there is just as great an opportunity for the constructive variety. So far, the Ministry has not attempted, in our view, to rush things through. Someone must give a lead, and whatever we think of the official efforts, they have at least provided this requirement. It is now necessary, and urgently necessary, to bring a united, non-Governmental, but nevertheless official, medical opinion to bear on all and each of these problems. Once again there is the crying need for bringing together the practical doctors of the country. A few days ago was published the first annual report of the Ministry's Chief Medical Officer, and this week we deal with the section referring to maternity and child welfare work. In a future issue we hope to review this important publication in greater detail. We have already referred on page 90 to one small factor in the unsatisfactory situation which at present exists.

### SUICIDE BY FASTING.

We publish on page 99 an article in which our contributor has collected with considerable care the actual medical facts concerning starvation and the length of time the human body can withstand it. It is a subject which has received no little popular attention of late, and the present circumstances, coupled with their political bearing, make it singularly expedient that the true scientific probabilities should be understood. It is an important point to note that mere lack of food substance, as such, has not the paramount importance that is generally imagined, the absence of vitamine, or accessory substance, intake being probably the more potent factor in physical decline.

### THE POSITION OF WOMEN STUDENTS.

Following upon our comments of last week upon the sex question in certain of the London Colleges, and particularly in those providing a medical training, we are in receipt of an article upon the corresponding question as it affects the University of Cambridge. Our correspondent, who writes as a past student and who has herself gained no little academic distinction, puts forward the women's case with a commendable degree of judgment and restraint. We publish her words in full on page 97, as it seems that this apparently burning question at Cambridge should, in course of its solution, provide valuable material and precedent upon which to build the future collegiate policy on co-education in our universities. The phases of the argument which directly affect medical training are undoubtedly of the more immediate interest to our readers, but the essential issues at stake concern all branches of professional education to a greater or lesser extent. We should welcome, therefore, correspondence on these matters, which are in need of the fullest ventilation, by those who have first-hand experience of modern university life.

### THE MYSTERIOUS STRANGER.

The story of the gentleman of "unpretentious demeanour and appearance" who deposits £500 notes in the offices of deserving charities is gaining momentum every day, and bids fair finally to equal the classic history of "A. B.," who behaved in a similar way some years ago. It is known that the mysterious benefactor has paid welcome visits to the Royal National Orthopædic Hospital, the Middlesex Hospital, the Royal Deaf and Dumb Institution, the Shipwrecked Fishermen and Mariners' Society, and two modest hospitals who do not wish any announcement on the subject, possibly having in view the consideration that if a hospital is thought too well off it will frighten away other givers. Anyhow, the visits of the benevolent stranger have apparently ceased for the moment. If he reads the newspapers he will be having plenty of amusement. The description by Mr. Morley, of the Orthopædic Hospital, of the gentleman's unpretentious personality is restrained, yet adequate in the circumstances. From this level of discretion to other levels is a far cry, and when we are told by one authority that he had on riding-breeches and top-boots and a "cup-final cloth cap," and by another that he wore a greasy suit and suffered from an inflamed eyelid, we wonder whether a desire for a mild sensation has not overridden the common rules of good taste and veracity.

### "OUR DAY" ALLOWED IN LEEDS.

The Leeds Watch Committee has reconsidered its decision to forbid a flag day for the British Red Cross and the Order of St. John. The origin of the early refusal was to limit the number of flag days, and in no case to grant the privilege to societies which did not enjoy it before the war. The date chosen was October 23, but the collections and efforts will be spread throughout the ensuing week. Leeds was the only town to refuse permission, and its second thoughts were therefore doubly welcome.

### DEATH OF HOSPITAL SECRETARY.

We regret to record the death of Mr. Arthur Compton Wickins, at the age of seventy, at Blackheath, on the 25th inst. Mr. Wickins was Secretary of the British Hospital for Mothers and Babies at Woolwich, and formerly Secretary of the British Lying-in Hospital, Endell Street, W.C., which was closed in 1913 and amalgamated with the Home for Mothers and Babies at Woolwich. Mr. Wickins was, before he left London, a well-known figure at hospital gatherings, and an active member of the Hospital Officers' Association.

### CLOSING OF AN HISTORIC WORKHOUSE.

Southwark Board of Guardians have closed St. George's Workhouse, in The Mint, Borough. The institution has a peculiar interest to all readers of the works of Charles Dickens, for here were depicted the scenes in the early life of Oliver Twist, where he asked for a second helping of gruel. The historic copper in which the gruel was prepared for the three daily meals of the inmates is to be offered to the Southwark Borough Council for preservation in the local museum. Vast changes have been made in Poor-Law administration since the days of which Dickens wrote, perhaps most of all in the medical administration. The birth of Oliver and the death of his mother, attended only by two old women, was an incident in workhouse life that would not be tolerated to-day by any Board of Guardians, nor would they permit a dying patient to be attended by the apprentice of the parish apothecary. Happily, these things are all changed for the better, though it is well to be reminded at times that there are still in existence some of the places so vividly depicted by the great novelist.

### BELFAST UNIVERSITY.

We note that, following the death of Sir John Byers, Professor of Midwifery, it has been resolved to establish two Chairs, instead of the existing one. The first will deal with midwifery and infantile diseases, and the second with gynæcology. It is proposed shortly to appoint professors to both these departments. The lectureship in economic history, vacant by the retirement of Mr. Conrad Gill, M.A., has been filled by the appointment of Mr. Joseph Lemberger, M.A., Trinity College, Oxford, who has already entered upon his duties. Also the Academic Council has decided that Sir William Whitla, M.P., should be granted the title of Emeritus Professor, in recognition of his long and valuable services as Professor of Materia Medica, of his many important contributions to the advancement of medical science, and of his signal services as M.P. for the Universtity.

### "A CURSE TO THE COUNTRY."

It seems that Mr. Philip Inman, the Secretary of the Hospital Saturday Fund, recently wrote to Sir Leo Chiozza Money and asked him to write an article for the *Saturday Fund Journal.* Sir Leo replied that he could not do this because he strongly believes that "the voluntary hospital system is a curse to the country." It goes without saying that Mr. Inman then supplied Sir Leo with a few particulars of the Saturday Fund, alluding to its democratic nature, and the evidence that it provided to show that the voluntary system is not repugnant to large numbers of working men. Mr. Inman did not, in our opinion, sufficiently dwell upon the educational side of hospital work, as represented by doctors, nurses, massage workers, and others, which must make a strong claim on the sympathy and support of all classes. It is very well known that there are some people who honestly consider that the voluntary system might in the public interest be replaced by a State or municipal system. We thoroughly respect their views and listen with attention to their arguments. To hold such views is one thing, to express them in intemperate language is another.

### EAST SUFFOLK AND IPSWICH HOSPITAL.

The accounts of the recent fête held in Christchurch Park, Ipswich, towards liquidating the revenue debt of the East Suffolk and Ipswich Hospital have just been published. This single-day fête produced the sum of £2,443, and a cheque for this amount has been handed over to the chairman of the hospital. Although this sum will not go very far towards wiping off the deficiency in the hospital's revenue account, which at the commencement of the financial year stood at five figures, it is evidently one of the best results ever achieved by a single-day fête in a town of the size of Ipswich, and speaks as well for its organisation as for the esteem in which the institution is held by the populace. The town of Ipswich is evidently proud of its hospital and of the courage and enthusiasm of the managers of that institution. There can scarcely be another general hospital in the country which has been hit harder, done more work, made greater extensions, and faced greater difficulties than the East Suffolk and Ipswich Hospital. At the outbreak of the war it had just completed an extension scheme to its out-patient and electrical departments, following on a scheme of structural improvements inside the institution, which had cost over £20,000. Practically all the workers in the town have now been organised to contribute on a regular basis of 2d. per week for men and 1d. or ½d. for women. The result is that the income from employees has been increased from £1,931 in 1913 to £5,180, and the subscriptions from the employers and the general subscribers from £2,212 to £3,758.

### CHANGES AT THE LONDON TEMPERANCE HOSPITAL.

The London Temperance Hospital has suffered severe loss by the death of its honoured chairman, the Rt. Hon. Sir T. Vezey Strong, K.C.V.O., K.B.E. For twenty-one years he brilliantly guided the affairs of the hospital, and was an outstanding figure in the public life of the City, as well as in the hospital world. At the meeting of the Board of Management on October 13 Major Richard Rigg, O.B.E., T.D., J.P., barrister-at-law, was unanimously elected chairman. Major Rigg takes the helm in difficult days, but he brings to his task many distinguished qualities, and is in the prime of life. After many years of faithful and devoted service as vice-chairman, Edward Crawshaw, Esq., has resigned his office, but, fortunately, not his membership. H. Ernest Wood, Esq., L.C.C., J.P., and W. W. Parkinson, Esq., have been elected vice-chairmen. Lady Strong has become a member—the first lady member—of the Board of Management.

### NEW RATES FOR THE R.A.M.C.

New daily rates of pay for the officers of the Army Medical Service and the R.A.M.C. are announced as follows and will have effect from April 1, 1920. Major-General, £4 15s.; captain, £1 7s.; captain (after six years' total service), £1 8s.; captain (after ten years' total service), £1 11s.; and lieutenant £1 2s. The rates for colonels, lieutenant-colonels and majors will remain unchanged. It is also announced that in regard to retired pay, the "service element" will be at the rate of £15 for each completed year of service in the case of officers of the A.M.S. and R.A.M.C., instead of at the rate of £150 for the first fifteen years' service) and £15 for each subsequent year. The rate of "rank element" and the maximum rates of retired pay admissible for each rank remain unchanged.

### HOSPITAL REOPENS WARD.

It is hoped that before Founders' Day, on November 2, the whole of the work of the National Hospital for the Paralysed and Epileptic, Queen Square, W.C., will be resumed, the financial situation having improved. Anxiety is still felt for the future of the work, as £100 a day has to be provided to meet the expenditure.

### NEW HOSPITAL BUILDING FOUND TOO DEAR.

When the Princess Mary visited the Royal Hospital and Home for Incurables, Putney, recently, the chairman informed Her Royal Highness that, owing to the abnormally high cost of building operations, the Board had been compelled to abandon for the time being the building of the new Nurses' Home, but that the Board had almost completed arrangements whereby two large houses in the immediate neighbourhood of the hospital are being bought and adapted for use as a temporary Nurses' Home. The two houses in question are 170 and 172 West Hill, Putney, and are exactly opposite to the institution. The necessary alterations are now well on the way, and it is hoped that at no distant date accommodation for at least fifty members of the nursing staff will be available.

### TWO SECRETARIAL APPOINTMENTS.

The Royal Victoria Hospital, Belfast, is advertising for a superintendent. The salary is £500 per annum, with free house, coal, and light. As only the money part of the emoluments counts for income-tax purposes, the post is a fairly well-paid one; but even at that it is not likely to attract many non-local people in view of the unsettled state of the district. One unusual feature is that the appointment is open to men up to fifty-five years of age, the retiring age being sixty-five. Another appointment advertised is that of assistant secretary in "a large London voluntary hospital," with a commencing salary of £500 a year. Preference is given to candidates between twenty-five and thirty-five, with a University degree, business capacity, knowledge of accounts, committee work, and secretarial duties, but hospital experience is not essential. We welcome this instance of an attempt to attract a good type of man to the administrative side of hospital work. As a rule, the grades below a secretary are so badly paid that the best assistants, if they have to wait too long for promotion, take up other lines.

### EAST MIDLAND BRANCH OF BRITISH HOSPITALS' ASSOCIATION.

A meeting has been held at the General Hospital, Nottingham, with the object of forming a branch of the British Hospitals' Association. Mr. H. Wade Deacon, chairman of the Council and chairman of the Royal Infirmary, Liverpool, addressed a gathering in which the hospitals of the East Midlands, comprising the counties of Rutland, Leicester, Derby, Northampton, and Lincoln, were represented. We hope that the recent endeavour to create branches will be successful, for, in spite of the useful annual conferences, the Association hitherto has lacked cohesion. The members of the Council are generally scattered, and men living as far apart as London and Glasgow cannot well act constantly together. The creation of local branches should reduce this difficulty and provide more regular channels of communication between the centre body and the separate institutions if an intermediate link in the form of a local branch can be secured. By this means also hospitals in the same area can act together. Co-ordination should become the rule rather than the exception, and the hands of the central authority be strengthened.

### THE STATE OF GOOLE ISOLATION HOSPITAL.

The members of the Goole Urban District Council seem to be agreed that the Goole Isolation Hospital is in a state of such dilapidation as to be fit rather for vermin than for men. One councillor declared that the beds were in a disgraceful state, and that rats had been found in them; and no one seemed to doubt the complete neglect into which the institution has fallen. Certainly no staff will be found until all this is altered, and certain repairs have been authorised and the General Purposes Committee empowered to purchase beds. In the condition described the building is worse than useless, and while we commend those, like Mr. Shaw, who wish it to be put in order, an inquiry should be made into the origin of the neglect. Whoever is responsible will have saddled the Council with considerable expense, and have defeated the object for which the hospital exists, since epidemics wait for no one.

### BASCHURCH HOSPITAL MOVES TO OSWESTRY.

The Shropshire Orthopædic Hospital, which used to be known as the Baschurch Surgical Home, has moved to Park Hall Camp, Oswestry. A gift of £25,000 from the Red Cross Society enabled the committee to buy the Military Hospital at Park Hall Camp for £18,000. The site cost £2,000, and £7,000 was spent on equipment. It is twenty years since the Baschurch Hospital was founded by Miss Hunt, a local resident, as a hospital for surgical tuberculosis. In its new home, the purchase of which owes much to Miss Hunt's energy, the hospital now consists of eighty buildings, standing on nineteen acres of land. Two hundred ex-Service patients and 150 children are about to be transferred to Oswestry, where the nursing staff numbers seventy-four, together with some fifty craftsmen and others. It was from small beginnings that the hospital has grown to its present size.

### DAYLIGHT DANGERS.

York City Medical Officer of Health, who in some of his annual reports has commented upon the insufficiency of sleep, now says, in a report to the City Council, that, in the opinion of many teachers and other observers of children, the daylight-saving system of the last three or four summers, so pleasant and valuable in many ways, is inimical to the educational and physical fitness of large numbers of them, because they are not sent to bed by the clock, but are allowed to play out in the streets and elsewhere until dusk. The consequence is, therefore, that during the summer many children lose an hour's sleep. The minimum number of hours of sleep per day for a young child should be twelve and for a child over ten years of age ten hours. Such inimical causes as he mentions cannot fail to inhibit the mentality of the children involved. The child half asleep, or hungry, or suffering from the vitiated air in an overcrowded room the night before, cannot possibly be expected to make normal progress at school.

### THE FUTURE OF THE CLINIC.

For the past year the old Birmingham Children's Hospital, known as the Broad Street clinic for the West Midland region, has been treating disabled men, mainly orthopædic cases, under the Ministry of Pensions. The special Pensions Board presides there and tropical and nervous diseases are also treated. The average attendances have been 2,500 a week, and the more difficult cases from the sub-clinics in the five central counties often come to Broad Street, where the laboratory work for the whole region is done. The day's work falls into three sessions, from 9 a.m. to 8 p.m., and this plan avoids much of the delay formerly complained of. For the convenience of the patients the S. John Ambulance Association runs a canteen, where the men can procure light refreshments at cost price. Beside massage and electric treatment, and the department for taking casts of deformed limbs, there is a gymnasium for regulated exercises. Add to these departments the sections devoted to ear, nose and throat treatment and some idea of the extent of the institution can be gained. The work done in these clinics, of which the Broad Street institution is the type, will perhaps be completed in another five years. By that time we hope the tradition of their usefulness will be so strongly established that a permanent use may be found for them for out-patients, whose special needs demand exactly this system and these resources. In that way the clinic will become a permanent part of our hospital system, and relieve the pressure on the out-patient departments of the general hospitals, which always have more work than they can do.

### AN ODD CONTRAST.

The behaviour of two patients on leaving the East Suffolk Hospital, Ipswich, makes an odd contrast. One lately sent ten Treasury notes in grateful remembrance of the treatment received by his late wife and daughter six years ago, and for kindness received when visiting them, "from a grateful working man." The other case is that of a prosperous-looking man who, after recovery from a serious accident, met a request for recompense for the cost of his treatment by offering to pay for the daily papers supplied to him! This offer was refused. But he gave the hospital one of those rare examples of ingratitude which is serviceable in its way. Hospitals never know with whom they may be dealing, and the worst as well as the best has a lesson for the general public.

### THIS WEEK'S DRUG MARKET.

It goes without saying that there is no improvement in business, but in view of the probability of a settlement of the miners' dispute there is a more hopeful spirit abroad, and it would not be surprising if and when a satisfactory agreement is arrived at there will be a general revival in trade. In the meantime prices still have a downward tendency, but it is well worth considering whether the present is not a favourable opportunity to make purchases. Those who feel convinced that we are on the eve of a settlement of labour troubles—for the time being—will probably take advantage of some of the bargains obtainable. To a large extent the steady downward movement of prices that has been in process for the last few months has been due to the absence of buying, and it is more than likely that when the demand sets in again prices will firm up, and that those who get in at the commencement will do pretty well. In consequence of a fall in the price of quicksilver, makers of calomel, corrosive sublimate and other salts of mercury have reduced their quotations. Several of the synthetic drugs have moved downward in price, and at the moment it should be possible to buy to advantage. Potassium bromide is again cheaper and supplies are plentiful. Benzoates, permanganate of potash, cocaine, isinglass, salol, and camphor are cheaper. The demand for eucalyptus oil has become quieter and prices are barely maintained. Various drugs are coming in more freely from Germany, and this fact should be considered in conjunction with the above remarks as to the advisability of buying.

